# Examination of SARS-CoV-2 In-Class Transmission at a Large Urban University With Public Health Mandates Using Epidemiological and Genomic Methodology

**DOI:** 10.1001/jamanetworkopen.2022.25430

**Published:** 2022-08-05

**Authors:** Kayla Kuhfeldt, Jacquelyn Turcinovic, Madison Sullivan, Lena Landaverde, Lynn Doucette-Stamm, Davidson H. Hamer, Judy T. Platt, Catherine Klapperich, Hannah E. Landsberg, John H. Connor

**Affiliations:** 1 Student Health Services, Boston University, Boston, Massachusetts; 2 Department of Microbiology, Boston University School of Medicine, Boston, Massachusetts; 3 National Emerging Infectious Diseases Laboratories, Boston University, Boston, Massachusetts; 4 Program in Bioinformatics, Boston University, Boston, Massachusetts; 5 Department of Biomedical Engineering and Precision Diagnostics Center, Boston University, Boston, Massachusetts; 6 Boston University Clinical Testing Laboratory, Research Department, Boston University, Boston, Massachusetts; 7 Department of Global Health, Boston University School of Public Health, Boston, Massachusetts; 8 Section of Infectious Disease, Department of Medicine, Boston University School of Medicine; Boston, Massachusetts; 9 Center for Emerging Infectious Disease Research and Policy, Boston University, Boston, Massachusetts

## Abstract

**Importance:**

SARS-CoV-2, the causative agent of COVID-19, has displayed person-to-person transmission in a variety of indoor situations. This potential for robust transmission has posed significant challenges and concerns for day-to-day activities of colleges and universities where indoor learning is a focus for students, faculty, and staff.

**Objective:**

To assess whether in-class instruction without any physical distancing, but with other public health mitigation strategies, is a risk for driving SARS-CoV-2 transmission.

**Design, Setting, and Participants:**

This cohort study examined the evidence for SARS-CoV-2 transmission on a large urban US university campus using contact tracing, class attendance, and whole genome sequencing during the 2021 fall semester. Eligible participants were on-campus and off-campus individuals involved in campus activities. Data were analyzed between September and December 2021.

**Exposures:**

Participation in class and work activities on a campus with mandated vaccination and indoor masking but that was otherwise fully open without physical distancing during a time of ongoing transmission of SARS-CoV-2, both at the university and in the surrounding counties.

**Main Outcomes and Measures:**

Likelihood of in-class infection was assessed by measuring the genetic distance between all potential in-class transmission pairings using polymerase chain reaction testing.

**Results:**

More than 600 000 polymerase chain reaction tests were conducted throughout the semester, with 896 tests (0.1%) showing detectable SARS-CoV-2; there were over 850 cases of SARS-CoV-2 infection identified through weekly surveillance testing of all students and faculty on campus during the fall 2021 semester. The rolling mean average of positive tests ranged between 4 and 27 daily cases. Of more than 140 000 in-person class events and a total student population of 33 000 between graduate and undergraduate students, only 9 instances of potential in-class transmission were identified, accounting for 0.0045% of all classroom meetings.

**Conclusions and Relevance:**

In this cohort study, the data suggested that under robust transmission abatement strategies, in-class instruction was not an appreciable source of disease transmission.

## Key Points

**Question:**

Is there evidence of in-class transmission of SARS-CoV-2 on a university campus that had mandated vaccination and masking?

**Findings:**

In this cohort study of 140 000 class meetings at a large US university, there were over 850 cases of SARS-CoV-2 infection identified through weekly surveillance testing of all students and faculty on campus during the fall 2021 semester. There were 9 instances of potential in-class transmission identified as identical lineages confirmed by SARS-CoV-2 genome sequencing, and none of these instances were confirmed to be in-class transmission.

**Meaning:**

These results suggest that in-class transmission of SAR-CoV-2 in an urban university with masking and vaccine protocols in-place was negligible.

## Teaser

This cohort study using surveillance testing data from a university located in a large US city examines incidence of in-classroom SARS-CoV-2 transmission on a college campus with contact tracing and vaccination and masking requirements.

## Introduction

SARS-CoV-2, the causative agent of COVID-19, has posed significant challenges to day-to-day activities of colleges and universities. Many institutions of higher education faced immense challenges in the 2020-2021 and 2021-2022 school years from spiking infections.^1^ With the vaccination rollout, infection risk lessened, and in-class instruction largely resumed. To help limit SARS-CoV-2 transmission, some institutions of higher education implemented vaccination requirements for students and faculty, along with nonpharmacological interventions such as surveillance testing, on-campus isolation of infected individuals, quarantine and frequent testing of close contacts, indoor masking, and enhancements to air filtration and circulation. These multifaceted interventions allowed in-class instruction to fully resume and dormitories to reopen and repopulate. Despite these efforts, transmission has been continuously present in campus life.

Understanding whether in-class instruction without any physical distancing is fueling transmission is an important question that has not yet been adequately addressed. Multiple theoretical calculations have suggested differing outcomes for vaccination or masking on in-class transmission,^2,3^ but evaluation of classroom transmission is minimal. Transmission in K-12 classrooms has shown to be minimal, but these studies lack comparability with lecture-size classrooms at the university level.^4,5,6^ The lack of such data has prompted uncertainty among students, faculty, and staff regarding the infection risk of in-person instruction. To address this evidence gap, we sought to unambiguously identify instances of SARS-CoV-2 transmission in the classroom environment at a large urban university. Using a blend of adaptive surveillance testing, traditional epidemiology, and viral genomics, we analyzed incidence of likely transmission over more than 140 000 class meetings.

## Methods

We linked campus-wide surveillance testing, contact tracing, and class rosters from September 1, 2021, to December 1, 2021. September 2021 marked the full return to in-person instruction at BU. There were more than 140 000 in-person class meetings during this time period. Class sizes ranged from 2 to over 400 individuals (mean class size, 31 students). Potential instances of in-class transmission were defined as 2 or more SARS-CoV-2 positive individuals who shared an in-person class but did not identify one another as close contacts outside of the classroom environment. Outside classroom exposures included, but were not limited to, social gatherings in dormitory or off campus, dining hall interactions, study groups or group projects, and athletic events. The Boston University (BU) Charles River Campus institutional review board reviewed and approved the protocol for sequencing to determine variant distribution and emergence on the BU campus and waived informed consent requirements based on study design. This study followed Strengthening the Reporting of Observational Studies in Epidemiology (STROBE) reporting guidelines for cohort studies.

### Public Health Policies

All BU buildings were inspected, received upgraded filtration when applicable, and had settings adjusted to increase frequency of fresh air exchanges. All mechanically ventilated classrooms had MERV-13 (minimum efficiency reporting values) filter upgrades and settings maximized to allow for increased fresh air and a minimum of 2 to 4 air circulations per hour. Non–mechanically ventilated classrooms had windows open when outdoor temperatures allowed and commercial-grade HEPA (high-efficiency particulate absorbing) filters placed within the rooms. Faculty had the option to remove their mask while lecturing if they were more than 6 feet from students and had increased their surveillance testing frequency. COVID-19 vaccination was mandated as of September 2, 2021, allowing for religious or medical exemption. Vaccination compliance at BU was defined as either completion of a SARS-CoV-2 vaccination series or a documented medical or religious exemption. BU students, faculty, and staff performed routine testing up to twice a week^7^ if considered a high contact group (ie, participant in school athletics, athletic trainers). Adaptive testing with an increased testing cadence was requested under circumstances seen to be high risk (eg, during outbreaks). Anterior nares samples were self-collected in sterile saline, and following clinical testing the residual discarded positive sample material was stored at −80 °C. For students not considered in a high-contact group, surveillance testing was an observed nasal swab done at a collection site once weekly where staff were present to show individuals how to properly perform the test and ensure the tests were properly handled.^8^ Adherence to testing was in excess of 95% in a student population of approximately 30 000. Compliance for testing, vaccinations, and isolation or quarantine status was monitored using a color-based badge system, which was a centralized system that limited access to campus buildings during isolation and quarantine periods. Enhanced air filtration efforts were practiced, including increased air changes per hour. In areas where there were not HVACs, standing air filters were used.

We identified all individuals with detectable SARS-CoV-2 by reverse transcriptase real-time polymerase chain reaction (rRT-PCR) using the US Centers for Disease Control and Prevention (CDC) primers for N1, N2, and Rnase P. Students who were infected with SARS-CoV-2 were placed in isolation status, or red badge status, for 10 days. Students who were exposed by someone who was infected with SARS-CoV-2 were advised on a quarantine timeline based on vaccination status and placed in quarantine status, or orange badge status.

### Contact Tracing

Case investigation and contact tracing were conducted for each case by BU contact tracers following adapted CDC and Massachusetts Department of Public Health (MDPH) protocols.^7^ In addition to traditional contact tracing, contact tracers utilized a Power BI (Microsoft) report to pull class rosters with classmate, faculty, and teaching assistant names to identify potential classroom crossover of confirmed cases. Contact tracing was used to identify potential at-work, in-class, or school-sponsored event transmission.

### SARS-CoV-2 Sequencing and Analysis

Residual discarded samples in sterile saline from SARS-CoV-2 positive swabs collected during the study window were used to amplify and sequence SARS-CoV-2 genomic RNA using the Illumina COVIDSeq Assay and sequenced on an Illumina NextSeq 500.^9^ Full-length genomes for each amplified sample were then assembled through alignment to the Wuhan-Hu-1 reference sequence (NC_045512.2) using Bowtie 2.^10^ Nucleotide substitutions, insertions, and deletions were identified with LoFreq.^11^ Lineage assignment for each genome was carried out using Pangolin.^12^ Sequencing data was completed for all surveillance tests weekly and shared with the contact tracing team for further investigation or, in the reverse, individuals with suspicion of in-class transmission were flagged for sequencing.

### Pairwise Comparison of SARS-CoV-2 Genomes

Genome relatedness was quantified by counting the number of nucleotide differences between genomes using a custom script. Each genome was modeled as a binary single nucleotide variant (SNV) vector spanning all nucleotide changes found at greater than 50% in the data set. Presence of an SNV in more than 50% of the aligned reads was coded as a 1; otherwise, zero. After pairwise comparison identical (0 differences), different (1 to 2 differences), and unrelated (3 or more differences) sequence sets were defined.

## Results

### Return to Class and Analysis Window

September 1, 2021, marked the full return to in-person instruction at BU. COVID-19 vaccination was mandated as of September 2, allowing for religious or medical exemption. Vaccination rates for BU faculty, staff, and students respectively were 98.5%, 93.5%, and 98.7%. Masking was mandatory during in-person instruction, but social distancing was not enforced. We analyzed SARS-CoV-2 transmission beginning September 1 to December 1, 2021, which was just prior to the end of term and the beginning of finals. During this time there were more than 140 000 in-person class meetings. Class sizes ranged from 2 to over 400 individuals (mean class size, 31 students).

### SARS-CoV-2 Test Positivity During the Fall 2021 Semester

**Figure 1.**
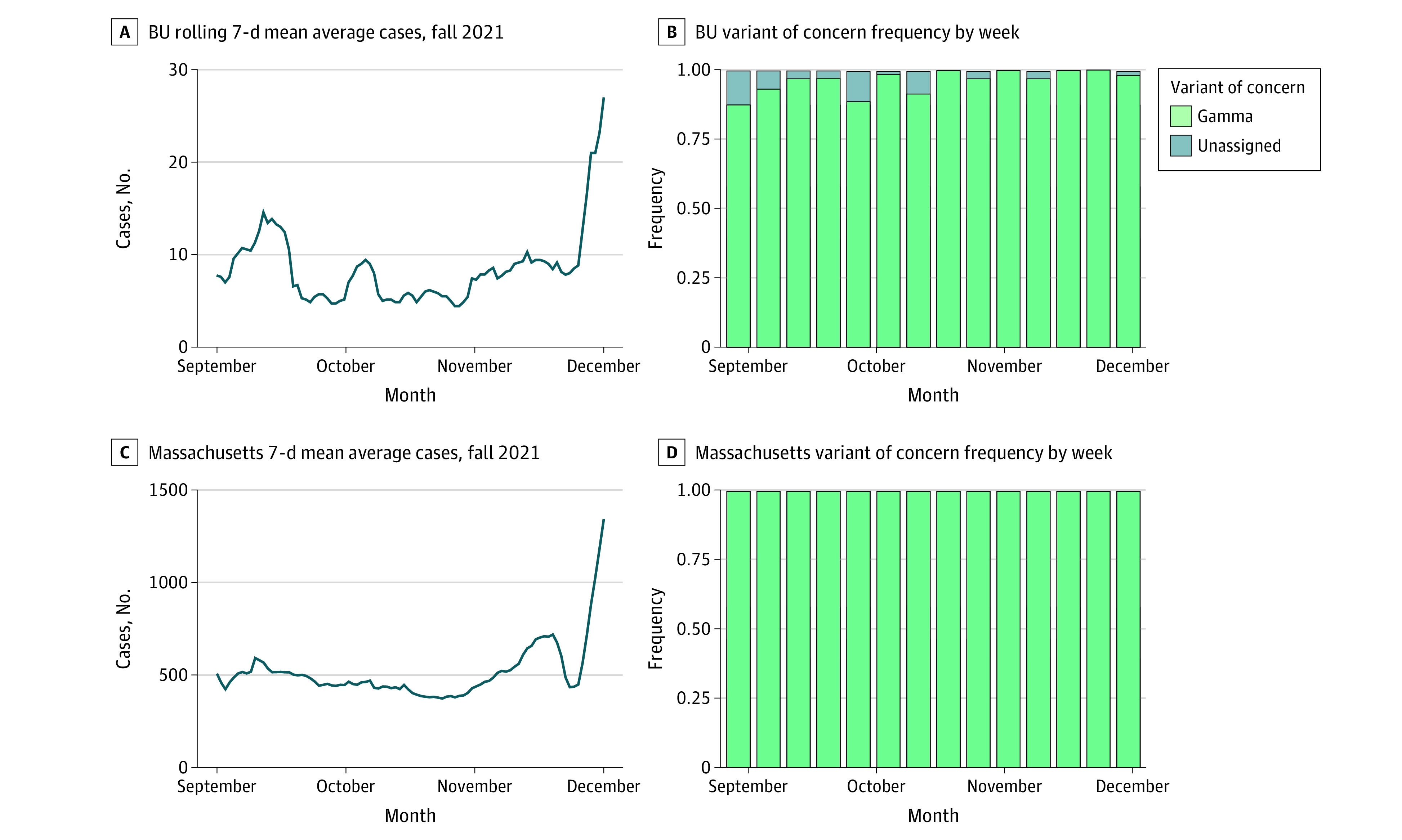
SARS-CoV-2 Positive Test Rates at Boston University (BU) and in Massachusetts Over the Study Period In the Massachusetts data in panel D, less than 1% non-Delta sequences were observed out of approximately 48 000 total cases (37 unassigned, 13 Omicron, 7 Mu, 5 Gamma, and 106 non–variant of concern).

There was significant SARS-CoV-2 infection throughout the semester. During our analysis period, there was weekly surveillance testing for SARS CoV-2 via rRT-PCR (anterior-nares based collection) at a Critical Laboratory Improvement Amendments–certified BU laboratory.^7^ All members of the BU community with an on-campus presence were tested. In total more than 600 000 SARS-CoV-2 PCR tests were conducted; of these approximately 896 (0.1%) of these tests showed detectable SARS-CoV-2. The 7-day rolling mean average ranged between 4 and 27 daily cases for most of the semester with a dramatic increase the last week of November (Figure 1). Whole SARS-CoV-2 genome sequencing was attempted on all PCR positive samples (success rate of 96.4%). This sequencing identified only Delta variant genomes circulating on campus.

### Disease Burden in the Surrounding Community

SARS-CoV-2 transmission in the surrounding environment was also significant over the study period. The 7-day rolling mean of SARS-CoV-2 positive cases in Massachusetts was between 374 and 1346 cases per day, with a rise in the last week of November to more than 1200 per day (Figure 1). The overwhelming majority of SARS-CoV-2 genome sequenced in Massachusetts over this time period were the Delta variant. These data illustrate sustained Delta variant transmission both within and outside the BU campus over the study period.

**Figure 2.**
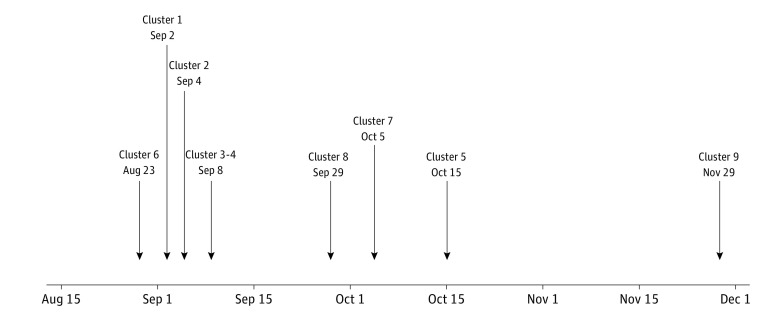
Identified Potential Incidents of Classroom Transmission Listed dates indicate approximate collection date for the first case in each cluster. See Table for more information on each cluster.

All 776 PCR positive samples were reported to the BU contact tracing team. The overwhelming majority of cases identified likely onto-campus (genome sequences not before identified on campus) transmission events associated with living arrangements (eg, roommates), socialization, or extended contact-time events. Of all PCR positive cases, a small number were deemed potential in-class transmission events. There were 9 instances of potential classroom transmission, accounting for approximately 0.0045% of all classroom meetings. These instances of potential classroom transmission did not involve more than 3 potential linked individuals and were not clustered in time (Figure 2). They were not associated with any individual class or individual attendance pattern.

For each potential in-classroom transmission event, we carefully analyzed the SARS-CoV-2 genomes. Each successfully sequenced genome was compared with all other genomes identified through surveillance testing. Samples with zero nucleotide differences across the genome (identical upon comparison) were deemed to be part of a linked transmission event based on known SARS-CoV-2 mutation rates and our history of validation through contact tracing. Genomes with 1 nucleotide difference were deemed to be possibly related, and those with 2 nucleotide changes were considered highly unlikely to be transmission events unless there was clear epidemiological support. Genomes that differed by 3 or more nucleotides across the SARS-CoV-2 genome were not considered to be linked.

**Figure 3.**
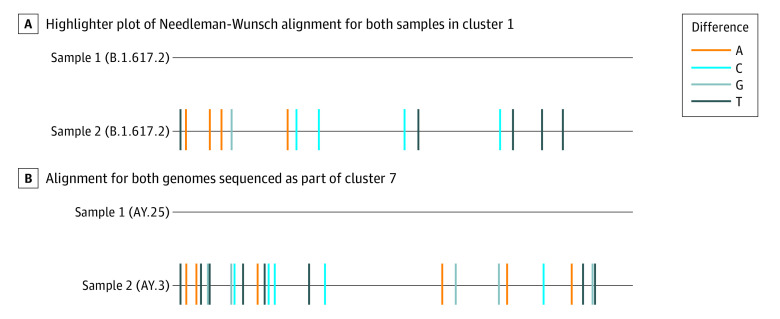
Sequence Comparison of SARS-CoV-2 Genomes From Potential In-Class Transmission Events Nucleotide changes were identified at the consensus level (≥50%) are highlighted according to the nucleotide difference identified in the second genome. Low-coverage ends were masked for comparison.

Seven of 9 potential transmission event pairs were identified as coming from different Pango lineages and as having more than 3 nucleotide differences (between 14 and 45 nucleotide differences). Thus, the genomic analysis suggested that they are unlikely to be classroom transmission events (Table). When comparing the genome sequences from the first potential classroom transmission, each of these genomes was also found to be genetically linked elsewhere (Figure 3; eFigure 1 in the Supplement). Sample 1 was identical to 6 other samples sequenced at BU/National Emerging Infectious Diseases Laboratories that were associated with an unrelated social event by contact tracing. Sample 2 was identical to another sequenced genome in the GISAID Initiative database from out of state. These comparisons strongly suggested that infection occurred outside the classroom environment.

**Table.** Instances of Potential Classroom-Based Transmission From September 1 to December 1, 2021

Potential cluster investigated	Date	No. of potential in-class transmission individuals	No. of samples sequenced	Genome lineage	No. of nucleotide differences between samples	Additional potential exposure points
1	Early September	2	2	AY.103 & B.1.617.2	14	Dining halls
2	Early September	2	2	AY.3 & B.1.617.2	42	Off-campus gathering, dorm crossover
3	Early September	2	1	B.1.671.2	Undetermined	
4	Early September	2	2	AY.4 & B.1.617.2	40	Boston bars, dental clinic
5	Mid-October	2	2	AY.25 & B.1.617.2	22	Dining hall, dorm crossover
6	Late August	2	2	AY.4 & B.1.617.2	23	Outside Boston locations
7	Early October	2	2	AY.3 & AY.25	26	Classroom building crossover
8	End September	3	3	AY.20	≤2	Public transportation, roommates, lunch
9	Late November	2	2	AY.103 & AY.25	22	Dining hall, building overlap

A second instance of potential transmission was from the middle of the study period (Figure 3; eFigure 2 in the Supplement). This potential transmission event also involved 2 individuals who did not note other potential sources of infection upon interview but whose viral genome sequences differed by more than 25 nucleotides. Sample 1 was 1 nucleotide away from more than 10 genomes available in GISAID that were sequenced through the Broad Institute, suggesting local transmission that was not classroom related. Sample 2 of this comparison was identical to more than 5 other genomes sequence through surveillance testing and was associated with a social event.

## Discussion

With the ongoing concern of safety for students, faculty, and staff as universities return to fully normal functioning, the use of epidemiology and genome sequencing is an effective model for understanding overall disease transmission safety in the classroom setting. Understanding the risk of classroom transmission to assess against the benefits of in-person learning is important for university leadership decision-making. In this university setting that had mask mandates in the classroom and a vaccine mandate for the university community, our data showed that that in-person learning was not contributing to the overall spread of SARS-CoV-2 in the campus community.

### Limitations

Limitations of our analysis included some inherent subjectivity in traditional epidemiologic investigations that center on an individual remembering all relevant interactions. This study occurred during a phase of the pandemic when the only circulating variants were associated with Delta sublineages, and the findings may not apply to other SARS-CoV-2 variants.

## Conclusions

With the ongoing concern of safety for students, faculty, and staff as universities return to fully functioning, an effective model for overall disease transmission safety in the classroom setting is necessary. Our data support the hypothesis that a combination of SARS-CoV-2 vaccination and risk mitigation measures including indoor masking, regular surveillance testing, and enhanced air filtration can be highly effective at limiting disease spread within a large university academic environment to the extent that classroom transmission risk is negligible.

## Notes

Supplement.
eMethods.
**eFigure 1.** Coverage and Mutation Profiles of Cluster 1 **eFigure 2.** Coverage and Mutation Profiles of Cluster 7 **eTable.** Key Statistics Regarding Classroom Meetings, Vaccination Rates, Non-pharmaceutical Interventions, and SARS-CoV-2+ Individuals Detected on Campus From September 1 to December 1, 2021
