# Supplementary material for: Examination of SARS-CoV-2 In-Class Transmission at a Large Urban University With Public Health Mandates Using Epidemiological and Genomic Methodology
Source: JAMA Netw Open. 2022 Aug 5;5(8):e2225430. doi: 10.1001/jamanetworkopen.2022.25430 (PMC9356317; doi:10.1001/jamanetworkopen.2022.25430)
Supplement: Supplement. — eMethods. eFigure 1. Coverage and Mutation Profiles of Cluster 1 eFigure 2. Coverage and Mutation Profiles of Cluster 7 eTable. Key Statistics Regarding Classroom Meetings, Vaccination Rates, Non-pharmaceutical Interventions, and SARS-CoV-2+ Individuals Detected on Campus From September 1 to December 1, 2021 [file jamanetwopen-e2225430-s001.pdf]

Supplemental Online Content

Kuhfeldt K, Turcinovic J, Sullivan M, et al. Examination of SARS-CoV-2 in-class transmission at a large urban university with public health mandates using epidemiological and genomic methodology. *JAMA Netw Open.* 2022;5(8):e2225430. doi:10.1001/jamanetworkopen.2022.25430

eMethods.

eFigure 1. Coverage and Mutation Profiles of Cluster 1

eFigure 2. Coverage and Mutation Profiles of Cluster 7

eTable. Key Statistics Regarding Classroom Meetings, Vaccination Rates, Non-pharmaceutical Interventions, and SARS-CoV-2+ Individuals Detected on Campus From September 1 to December 1, 2021

This supplemental material has been provided by the authors to give readers additional information about their work.

© 2022 Kuhfeldt K et al. *JAMA Network Open.*

eMethods.

After alignment to the Wuhan Hu-1 RefSeq (see main methods), samtools was used to generate a table of aligned reads per nucleotide. This table was plotted with a log10 y-axis to generate coverage traces for samples in clusters #1 (Supp. Fig. 1A) and #7 (Supp. Fig. 2A). The same alignment file was passed to Lofreq, and the resulting VCF file was used to calculate the percent of mutated reads per nucleotide (Supp. Fig. 1B; Supp. Fig. 2B). VCFs were then filtered to retain only nucleotide changes present in the consensus sequence (>50% of aligned reads). Consensus changes found in both cluster #1 samples (Supp. Fig. 1C) or in both cluster #7 samples (Supp. Fig. 2C) were then artificially set to 0% to visualize the differences between samples.

© 2022 Kuhfeldt K et al. *JAMA Network Open.*

eFigure 1. Coverage and Mutation Profiles of Cluster 1

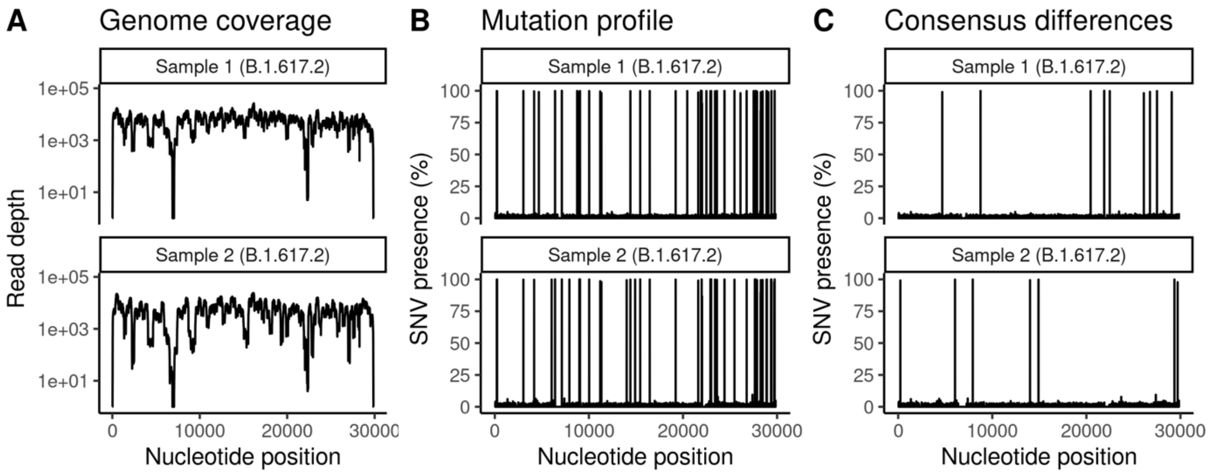

Both samples had >4000X median aligned read depth (A). The full mutational profiles of both samples in the cluster can be seen in (B). The differences between the samples are visualized in (C), where mutations found in both samples have been artificially set to 0%.

© 2022 Kuhfeldt K et al. *JAMA Network Open.*

eFigure 2. Coverage and Mutation Profiles of Cluster 7

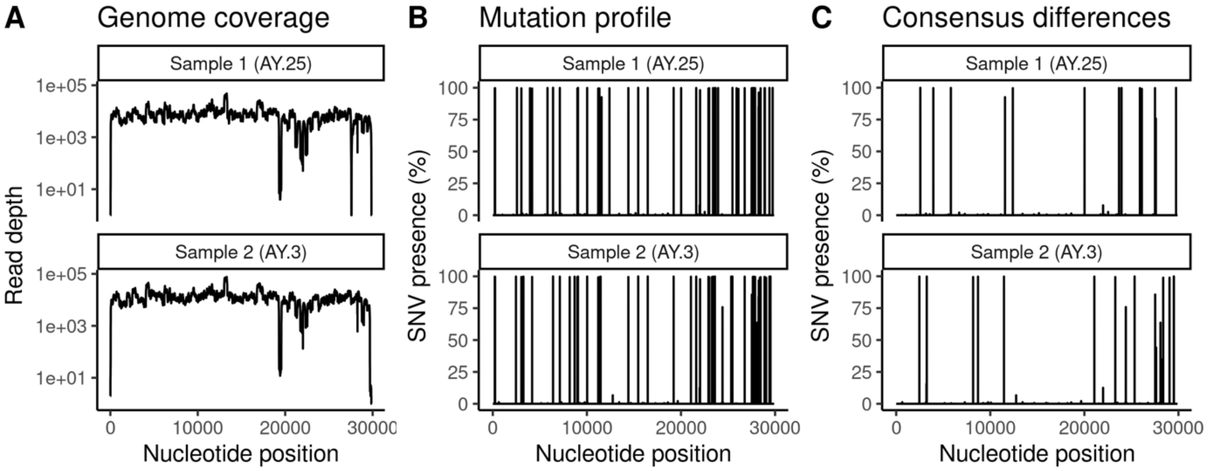

Both samples had >6000X median aligned read depth (A). The full mutational profiles of both samples in the cluster can be seen in (B). The differences between the samples are visualized in (C), where mutations found in both samples have been artificially set to 0%.

© 2022 Kuhfeldt K et al. *JAMA Network Open.*

eTable. Key Statistics Regarding Classroom Meetings, Vaccination Rates, Non-pharmaceutical Interventions, and SARS-CoV-2+ Individuals Detected on Campus From September 1 to December 1, 2021

Time Period	*# of Class Meetings*	*% Students Vaccinated*	*% Faculty Vaccinated*	*Testing Freq*	*Masking Policy*	*Typical Classroom Air Exch*	*SARS-CoV-2+ Individuals*
Aug 1-Dec 1	>140,000	98.7%	98.8%	Once Weekly	Required In-Class	4-6 eACH/h Merv13 minimum	776

Abbreviations: eACH- effective air changes, Freq-frequency, Exch-exchanges.

© 2022 Kuhfeldt K et al. *JAMA Network Open.*
